# Next-generation sequencing analysis of the molecular spectrum of thalassemia in Southern Jiangxi, China

**DOI:** 10.1186/s40246-023-00520-5

**Published:** 2023-08-17

**Authors:** Tong Yang, Xuemei Luo, Yanqiu Liu, Min Lin, Qinfei Zhao, Wenqian Zhang, Zhigang Chen, Minghua Dong, Junli Wang, Qi Wang, Xiaokang Zhang, Tianyu Zhong

**Affiliations:** 1 The First School of Clinical Medicine, Gannan Medical University, Ganzhou, China; 2 Laboratory Medicine, First Affiliated Hospital of Gannan Medical University, Ganzhou, 341000 Jiangxi China; 3 Ganzhou Municipal Health Commission, Ganzhou, China; 4 Department of Medical Genetics, Jiangxi Maternal and Child Health Hospital, Nanchang, China; 5 School of Food Engineering and Biotechnology, Hanshan Normal University, Chaozhou, China; 6 BGI Genomics, BGI-Shenzhen, Shenzhen, China; 7 BGI-Wuhan Clinical Laboratories, BGI-Shenzhen, Wuhan, China; 8 School of Public Health and Health Management, Gannan Medical University, Ganzhou, China; 9 Affiliated Hospital of Youjiang Medical University for Nationalities, Baise, China

**Keywords:** Next-generation sequencing, Thalassemia, Molecular spectrum, China

## Abstract

**Background:**

Thalassemia is an extremely prevalent monogenic inherited blood disorder in southern China. It is important to comprehensively understand the molecular spectrum of thalassemia in an area with such a high prevalence of thalassemia before taking appropriate actions for the prevention and treatment of this disorder. Herein, we explored the clinical feasibility of using next-generation sequencing (NGS) for large-scale population screening to illustrate the prevalence and spectrum of thalassemia in Southern Jiangxi.

**Methods:**

Blood samples collected from 136,312 residents of reproductive age in Southern Jiangxi were characterized for thalassemia by NGS. A retrospective analysis was then conducted on blood samples determined to be positive for thalassemia.

**Results:**

In total, 19,827 (14.545%) subjects were diagnosed as thalassemia carriers, and the thalassemia prevalence rate significantly varied by geographical region (*p* < 0.001). A total of 40 α-thalassemia genotypes including 21 rare genotypes were identified, with -@-^SEA^/αα being the most prevalent genotype. 42 β-thalassemia genotypes including 27 rare genotypes were identified, with the most common mutation IVS II-654 C > T accounting for 35.257% of these β-thalassemia genotypes. Furthermore, 74 genotypes were identified among 608 individuals with combined α- and β-thalassemia. Notably, most individuals with rare thalassemia mutations had mildly abnormal hematologic parameters including microcytic hypochromia.

**Conclusions:**

Our findings demonstrate the great heterogeneity and diverse spectrum of thalassemia in Southern Jiangxi, emphasizing the importance and necessity of persistent prevention and control of thalassemia in this region. Additionally, our findings further suggest that NGS can effectively identify rare mutations and reduce the misdiagnosis rate of thalassemia.

**Supplementary Information:**

The online version contains supplementary material available at 10.1186/s40246-023-00520-5.

## Introduction

Thalassemia is a group of hereditary blood diseases caused by a defect in the globin gene which leads to reduced or even a complete absence of globin peptide chains used for forming hemoglobin, eventually resulting in clinical symptoms, such as chronic hemolysis and anemia [1]. According to the types of globin involved, thalassemia can be classified into α-, β-, δ-, and γ-thalassemia [2]. Thalassemia is mainly manifested as chronic progressive hemolytic anemia. The degree of anemia varies depending on the type and amount of hemoglobin synthesized. Thalassemia minor may manifest as mild anemia or present in a patient with no clinical symptoms, while thalassemia major often leads to severe anemia [1, 3]. Hemoglobinopathies are widely prevalent in Mediterranean coastal areas, Africa, the Middle East, Southeast Asia, and southern China [4, 5] and pose significant public health problems and burdens on the communities in these areas. Thus, a comprehensive illustration of the prevalence and genotype distribution of this disease is indispensable for the prevention and control of thalassemia. Early diagnosis of thalassemia is conducive to timely prevention and treatment of severe thalassemia [6]. Hitherto, thalassemia carrier screening and genetic counseling have been demonstrated to be the most effective solutions to reduce thalassemia major [7, 8].

**Fig. 1:**
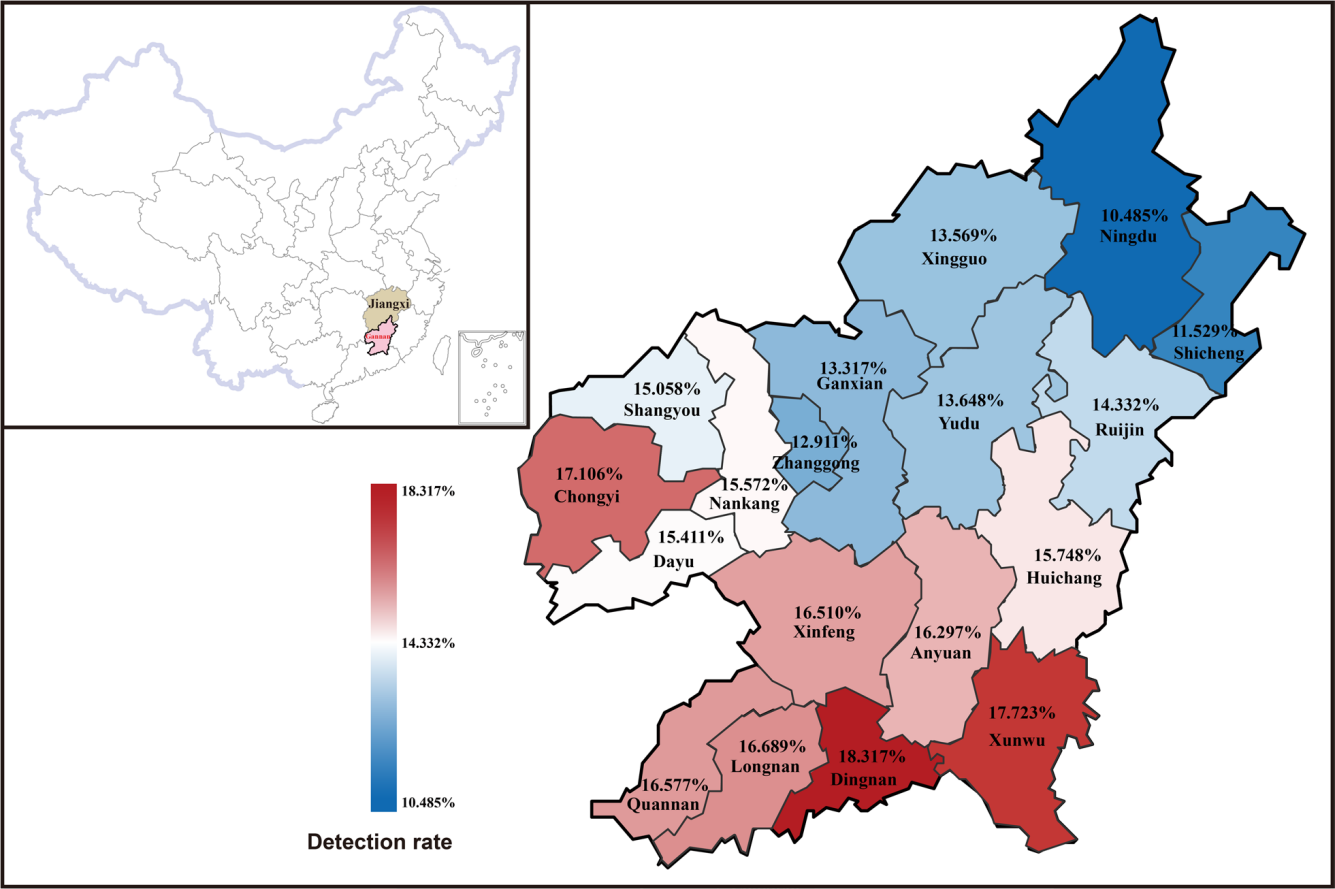
Detection rate of thalassemia and its distribution in Southern Jiangxi, China

Previous studies have shown that thalassemia in China is mainly distributed in southern regions, with the highest thalassemia carrier rate occurring in Guangxi Province [9]. Jiangxi Province consists of 11 cities with a total population of 45,188,600 spread over an area of 166,900 km^2^ (http://www.gztj.gov.cn), and it borders the province of Anhui to the north, Zhejiang to the northeast, Fujian to the east, Guangdong to the south, Hunan to the west, and Hubei to the northwest (Top left thumbnail of Fig. 1). While thalassemia has been reported to be highly prevalent in these neighboring provinces, including 16.450% in Guangdong [10], 10.780% in Hunan [11], and 6.800% in Fujian [12], Jiangxi has also been reported to have a total prevalence of 2.600% [13]. Ganzhou city, also known as the Gannan region, the southernmost city in Jiangxi Province, is the main gathering place of the Hakka people, and it had been reported that the carrier rate of thalassemia in this city was as high as 9.490% [14]. However, previous studies have had some limitations due to their small sample sizes or the use of nonrepresentative populations [13, 14]. The majority of the subjects enrolled were children or adults who visited hospitals for the diagnosis of various diseases. At present, integration of reverse dot blot (RDB), gap-PCR, and fluorescence PCR melting curve is the most commonly used method in identifying thalassemic mutations [15, 16]. The major limitation of these methods is that they only identify common variations. Additionally, the thalassemia in the Gannan region population has not yet been investigated using a large-scale and comprehensive epidemiological survey [14]. Therefore, the spectrum of thalassemic variations in this region has not been comprehensively explored. Taken together, it is reasonable to assume that many types of mutations in thalassemia may have been overlooked in previous studies.

Next-generation sequencing (NGS) enables rapid and high-throughput detection of genetic variants [17]. Recently, technologies such as whole genome sequencing (WGS), exome sequencing, or targeted enrichment panel sequencing have been widely applied in the molecular diagnosis of various genetic disorders [18, 19]. Herein, we used NGS for the first time to analyze thalassemia distribution in 136,312 subjects of reproductive age enrolled from April 2019 to April 2021 in the Gannan region, which provides a theoretical basis for the screening, prevention, and treatment of thalassemia in other regions. Moreover, our findings demonstrated that NGS could be effectively used to identify rare mutations undetectable using traditional testing methods, potentially further reducing the misdiagnosis rate of thalassemia.

## Materials and methods

### Participants

This study was approved by the Ethics Committee of the First Affiliated Hospital of Gannan Medical University. During the period between April 2019 and April 2021, a total of 75,955 couples, with at least one being a resident of the Southern Jiangxi region (Fig. 1), participated in the “Implementation Plan for the Free Gene Detection of Thalassemia in Ganzhou City (2019–2022)”. A total of 136,312 subjects of Ganzhou origin were screened from these couples, including those of Han nationality (99.244%) or She and Hui nationalities (0.756%). The age distribution ranged from 18 to 54 years, with an average age of 26 years, and all individuals provided written informed consent prior to study enrollment. This study was also approved by the Ganzhou Municipal Health Commission and was conducted in accordance with the ethical guidelines for research on human subjects.

### Genomic DNA extraction

Genomic DNA was extracted from 200 μL whole blood samples using the QIAamp DNA Blood Mini Kit (Qiagen, Hilden, Germany). DNA extracts were then arrayed in 96-well plates, and concentration were quantified using a Qubit 3.0 Fluorometer (Thermo Fisher Scientific, Waltham, MA, USA). We required all samples to have a DNA concentration > 10 ng/mL and an A260/A280 ratio between 1.8 and 2.0 for downstream use.

### Thalassemia detection

A combined strategy of Gap-PCR and NGS was applied to detect thalassemia [11]. In brief, seven deletions were analyzed by Gap-PCR. Other mutations in globin genes were analyzed by NGS. Firstly, the full-length *HBA1*, *HBA2*, and *HBB* genes were amplified by PCR, resulting in the amplicons that spanned all the exons and introns of these three genes. Sequencing libraries were then constructed according to the MGISEQ-2000 sequencing library preparation protocol. Paired‐end (100 bp) ‐sequencing on the MGISEQ-2000 sequencer was used for generating sequencing data [19].

### Hematological analysis

First, 2 mL of peripheral venous blood samples from 30,995 subjects were collected using ethylene diamine tetraacetic acid K2 (EDTA-K_2_) anticoagulated tubes. Red cell indices were then determined using a SYSMEX XN1000 automatic blood cell analyzer (Kobe, Japan). Subjects with a low red blood cell (RBC) mean corpuscular volume (MCV) < 82 fl and/or mean corpuscular hemoglobin (MCH) < 27 pg were considered positive for thalassemia. Subjects with an MCV ≥ 82 fl and an MCH ≥ 27 pg were considered negative.

### Thalassemia genotype definition

Common α-thalassemia and β-thalassemia mutations were found to be prevalent in southern Chinese populations. Moreover, these mutations can often be identified using routine laboratory testing [20]. *HBA* and *HBB* genotype categories were defined in Additional file 1: Table S1.

### Data analysis and statistics

Statistical analysis was conducted using SPSS 23.0 software. A Chi-square (χ^2^) test was used to evaluate the differences in detection rates (α-thalassemia, β-thalassemia, and combined α-/β-thalassemia) between different regions and genders. *P* < 0.05 were considered significant.

## Results

### Prevalence of thalassemia in the Gannan region

A total of 136,312 subjects were enrolled in this study, including 71,093 males and 65,219 females. Of these, we identified 19,827 (14.545%) thalassemia carriers, which consisted of 14,298 (10.489%) cases of α-thalassemia, 4921 (3.610%) cases of β-thalassemia, and 608 (0.446%) cases of combined α-/β-thalassemia (Additional file 1: Table S2). Figure 1 shows the prevalence of thalassemia in different regions.

Dingnan presented the highest detection rate at 18.317% (442/2,413), which was significantly higher than that [10.485% (1138/10,854)] in Ningdu (χ^2^ = 115.451, *p* < 0.001), which had the lowest rate. However, no significant correlations were observed between gender and the detection rates of α-thalassemia, β-thalassemia, or combined α-/β-thalassemia (χ^2^ = 1.377, *p* = 0.241).

### Distribution of α-thalassemia genotypes

Among the 14,298 individuals with α-thalassemia mutations, 206 cases (1.441%) of Hb H disease (α^+^/α^0^) and 14,092 cases (98.559%) of α-thalassemia gene carriers (α^+^/α or α^+^/α^+^ or α^0^/α) were detected. We identified 40 distinct genotypes, including 11 deletions in 13,240 cases, 16 nondeletion mutations in 994 cases, and 13 deletions combined with nondeletion mutations in 64 cases. In addition, 159 cases of 21 rare gene types were also identified. -@-^SEA^/αα was the most abundant genotype, accounting for 54.105% of all α-thalassemia genotypes. Other genotypes, such as -α^3.7^/αα, -α^4.2^/αα, α^WS^α/αα, α^CS^α/αα, and α^QS^α/αα, also occurred frequently and represented 28.011%, 8.687%, 3.546%, 1.755%, and 0.902% of the studied population, respectively. It is worth noting that the top six abundant genotypes accounted for 97.007% of all the α-thalassemia cases (Table 1).

**Table 1:** Prevalence rate of α-thalassemia genotypes, phenotypes, and constituent ratios in Gannan populations

Genotype	Phenotype	Cases	Detection rate (%)	Constituent ratio (%)
-@-^SEA^/αα	α^0^/α	7736	5.675	54.105
-α^3.7^/αα	α^+^/α	4005	2.938	28.011
-α^4.2^/αα	α^+^/α	1242	0.911	8.687
α^WS^α/αα	α^+^/α	507	0.372	3.546
α^CS^α/αα	α^+^/α	251	0.184	1.755
α^QS^α/αα	α^+^/α	129	0.095	0.902
-α^3.7^/-@-^SEA^	α^+^/α^0^	119	0.087	0.832
Hb Phnom Penh/αα*	α^+^/α	59	0.043	0.413
-α^4.2^/-@-^SEA^	α^+^/α^0^	43	0.032	0.301
-@-^THAI^/αα*	α^0^/α	35	0.026	0.245
-α^3.7^/-α^3.7^	α^+^/α^+^	27	0.020	0.189
-α^3.7^/-α^4.2^	α^+^/α^+^	22	0.016	0.154
Init CD ATG > A-G /αα*	α^+^/α	17	0.012	0.119
α^WS^α/-@-^SEA^	α^+^/α^0^	17	0.012	0.119
CD 30 -GAG [-Glu]/αα*	α^+^/α	10	0.007	0.070
α^CS^α/-@-^SEA^	α^+^/α^0^	10	0.007	0.070
-α^4.2^/-α^4.2^	α^+^/α^+^	7	0.005	0.049
α^CS^α/-α^3.7^	α^+^/α^+^	8	0.006	0.056
α^WS^α/-α^3.7^	α^+^/α^+^	7	0.005	0.049
Hb Phnom Penh/-@-^SEA*^	α^+^/α^0^	6	0.004	0.042
CD 61 AAG > TAG [Lys > STOP]/αα*	α^+^/α	4	0.003	0.028
CD 109 (-C)/αα*	α^+^/α	3	0.002	0.021
-α^4.2^/-@-^THAI*^	α^+^/α^0^	3	0.002	0.021
α^QS^α/-@-^SEA^	α^+^/α^0^	3	0.002	0.021
α^QS^α/-α^3.7^	α^+^/α^+^	3	0.002	0.021
HKαα/-@-^SEA*^	α^+^/α^0^	3	0.002	0.021
CD 22–26 (-9 bp)/-α^3.7*^	α^+^/α^+^	2	0.001	0.014
α^fusion^/αα*	α^+^/α	5	0.004	0.035
α^WS^α/-α^4.2^	α^+^/α^+^	2	0.001	0.014
α^WS^α/Hb Phnom Penh*	α^+^/α^+^	1	0.001	0.007
Hb Phnom Penh/-α^3.7*^	α^+^/α^+^	1	0.001	0.007
CD 6 GAC > CAC [Asp > His]/αα*	α^+^/α	1	0.001	0.007
CD 32 ATG > ATA [Met > Ile]/αα*	α^+^/α	3	0.002	0.021
CD 17 GTC > TTC [Val > Phe]/αα*	α^+^/α	1	0.001	0.007
Init CD ATG > GTG/αα*	α^+^/α	1	0.001	0.007
IVS I-116 A > G/αα*	α^+^/α	1	0.001	0.007
IVS I-55 G > A/αα*	α^+^/α	1	0.001	0.007
-α^3.7^/-@-^THAI*^	α^+^/α^0^	1	0.001	0.007
α^QS^α/-α^4.2^	α^+^/α^+^	1	0.001	0.007
HKαα/-α^4.2*^	α^+^/α^+^	1	0.001	0.007
Total		14,298	10.489	100.000

*Indicates genotypes with rare mutations

### Distribution of β-thalassemia genotypes

A total of 4921 subjects with β-thalassemia were identified in this study, including 4910 (99.776%) heterozygotes, 2 (0.041%) mutant homozygotes, and 9 (0.183%) compound heterozygotes. In this cohort, we also found 42 distinct genotypes, including 27 rare genotypes. IVS II-654 C > T/β^N^ was the most prevalent genotype, accounting for 35.257% of all β-thalassemia genotypes. Other main genotypes included CD 41/42 (− CTTT)/β^N^, − 28 (A > G)/β^N^, CD 17 AAG > TAG [Lys > STOP]/β^N^, − 50 G > A/β^N^, 5'UTR + 43 to + 40 (− AAAC)/β^N^, and CD27-28 (+ C)/β^N^. Overall, these seven genotypes accounted for 91.425% of all detected β-thalassemia genotypes in this study (Table 2). Interestingly, − 50 (G > A)/β^N^ and 5'UTR + 43 to + 40 (− AAAC)/β^N^ have been shown to be rare β-thalassemia genotypes but accounted for a high proportion of patients at 3.190% and 4.125%, respectively.

**Table 2:** Prevalence rate of β-thalassemia genotypes, phenotypes and constituent ratios in Gannan populations

Genotype	Phenotype	Cases	Detection rate (%)	Constituent ratio (%)
IVS II-654 C > T/β^N^	β^+^/β^N^	1735	1.273	35.257
CD 41/42 (− CTTT)/β^N^	β^0^/β^N^	1396	1.024	28.368
− 28 (A > G)/β^N^	β^+^/β^N^	595	0.436	12.091
CD 17 AAG > TAG [Lys > STOP]/β^N^	β^0^/β^N^	299	0.219	6.076
− 50 (G > A)/β^N*^	β^+^/β^N^	157	0.115	3.190
5'UTR + 43 to + 40 (− AAAC)/β^N*^	β^+^/β^N^	203	0.149	4.125
CD27-28 (+ C)/β^N^	β^0^/β^N^	114	0.084	2.317
CD71-72 (+ A)/β^N^	β^0^/β^N^	69	0.051	1.402
Chinese ^G^γ + (^A^γδβ)^0^/β^N*^	β^0^/β^N^	62	0.045	1.260
CD 43 (GAG > TAG)/β^N^	β^0^/β^N^	31	0.023	0.630
CD14-15 (+ G)/β^N^	β^0^/β^N^	28	0.021	0.569
SEA-HPFH/β^N*^	β^0^/β^N^	27	0.020	0.549
− 90 (C > T)/β^N*^	β^+^/β^N^	26	0.019	0.528
IVS II-761 A > G/β^N*^	β^0^/β^N^	25	0.018	0.508
− 29 (A > G)/β^N^	β^+^/β^N^	22	0.016	0.447
CAP + 8 (C > T)/β^N*^	β^+^/β^N^	26	0.019	0.528
Taiwanese/β^N*^	β^0^/β^N^	14	0.010	0.284
CAP + 22 (G > A)/β^N*^	β^+^/β^N^	13	0.010	0.264
CD 26 GAG > AAG [Glu > Lys]/β^N^	β^+^/β^N^	35	0.026	0.711
− 72 (T > A)/β^N*^	β^+^/β^N^	4	0.003	0.081
CD 56–60 (+ 14 bp)/β^N*^	β^0^/β^N^	7	0.005	0.142
− 28 (A > C)/β^N^	β^+^/β^N^	3	0.002	0.061
− 30 (T > C)/β^N^	β^0^/β^N^	4	0.003	0.081
IVS II-848 C > T/β^N*^	β^+^/β^N^	3	0.002	0.061
− 28 (A > G)/ − 28 (A > G)	β^+^/β^+^	2	0.001	0.041
− 86 (C > A)/β^N*^	β^+^/β^N^	2	0.001	0.041
IVS II-613 (C > T)/β^N*^	β^+^/β^N^	3	0.002	0.061
− 50 (G > A)/ − 28 (A > G)*	β^+^/β^+^	1	0.001	0.020
− 50 (G > A)/ CD 17 AAG > TAG [Lys > STOP]	β^+^/β^0^	1	0.001	0.020
− 50 (G > A)/ CD 41/42 (− CTTT)*	β^+^/β^0^	1	0.001	0.020
5'UTR + 43 to + 40 (− AAAC)/ CD 41/42 (− CTTT)*	β^+^/β^0^	2	0.001	0.041
CAP + 8 (C > T)/ IVS II-654 C > T*	β^+^/β^+^	1	0.001	0.020
IVS II-654 C > T/Taiwanese*	β^+^/β^0^	1	0.001	0.020
− 56 (G > C)/β^N*^	β^+^/β^N^	1	0.001	0.020
+ 20 (C > T)/β^N*^	β^+^/β^N^	1	0.001	0.020
− 87 (C > T)/β^N*^	β^+^/β^N^	1	0.001	0.020
Chinese ^G^γ + (^A^γδβ)^0^/ − 28 (A > G)*	β^0^/β^+^	1	0.001	0.020
Chinese ^G^γ + (^A^γδβ)^0^/IVS II-654 C > T*	β^0^/β^+^	1	0.001	0.020
Codon 121 (G > T)/β^N*^	β^0^/β^N^	1	0.001	0.020
Init CD ATG > AGG/β^N^	β^0^/β^N^	1	0.001	0.020
IVS-I-129 (A > G)/β^N*^	β^0^/β^N^	1	0.001	0.020
IVS-I-5 (G > C)/β^N^	β^+^/β^N^	1	0.001	0.020
Total		4921	3.610	100.000

*Indicates genotypes with rare mutations

### Distribution of combined α-/β-thalassemia genotypes

We identified a total of 608 cases of combined α-/β-thalassemia, at a prevalence rate of 0.446% (608/136,312) in this group, which consisted of 74 genotypes. Among these genotypes, -@-^SEA^/αα combined with IVS II-654 C > T/β^N^ was the most frequent genotype (19.572%, 119/608), followed by -@-^SEA^/αα combined with codons CD 41/42 (− CTTT)/β^N^ (16.941%, 103/608), -α^3.7^/αα combined with IVS II-654 C > T/β^N^ (11.513%, 70/608), -α^3.7^/αα combined with CD 41/42 (− CTTT)/β^N^ (8.717%, 53/608), and -@-^SEA^/αα combined with − 28 (A > G)/β^N^ (6.579%, 40/608) (Table 3).

**Table 3:** Prevalence rate of combined α-/β-thalassemia genotypes, phenotypes and constituent ratios in Gannan populations

α-Thalassemia genotype	β-Thalassemia genotype	Phenotype	Cases	Detection rate (%)	Constituent ratio (%)
-@-^SEA^/αα	IVS II-654 C > T/β^N^	α^0^/α** + **β^+^/β^N^	119	0.087	19.572
-@-^SEA^/αα	CD 41/42 (− CTTT)/β^N^	α^0^/α** + **β^0^/β^N^	103	0.076	16.941
-α^3.7^/αα	IVS II-654 C > T/β^N^	α^+^/α** + **β^+^/β^N^	70	0.051	11.513
-α^3.7^/αα	CD 41/42 (− CTTT)/β^N^	α^+^/α** + **β^+^/β^N^	53	0.039	8.717
-@-^SEA^/αα	− 28 (A > G)/β^N^	α^0^/α** + **β^+^/β^N^	40	0.029	6.579
-@-^SEA^/αα	CD 17 AAG > TAG [Lys > STOP]/β^N^	α^0^/α** + **β^0^/β^N^	23	0.017	3.783
-α^4.2^/αα	IVS II-654 C > T/β^N^	α^+^/α** + **β^+^/β^N^	22	0.016	3.618
-α^4.2^/αα	CD 41/42 (− CTTT)/β^N^	α^+^/α** + **β^0^/β^N^	15	0.011	2.467
-α^3.7^/αα	− 28 (A > G)/β^N^	α^+^/α** + **β^+^/β^N^	12	0.009	1.974
-α^3.7^/αα	CD 17 AAG > TAG [Lys > STOP]/β^N^	α^+^/α** + **β^0^/β^N^	11	0.008	1.809
α^WS^α/αα	CD 41/42 (-CTTT)/β^N^	α^+^/α** + **β^0^/β^N^	9	0.007	1.480
α^QS^α/αα	IVS II-654 C > T/β^N^	α^+^/α** + **β^+^/β^N^	8	0.006	1.316
-α^4.2^/αα	− 28 (A > G)/β^N^	α^+^/α** + **β^+^/β^N^	7	0.005	1.151
α^WS^α/αα	IVS II-654 C > T/β^N^	α^+^/α** + **β^+^/β^N^	7	0.005	1.151
-@-^SEA^/αα	CD27-28 (+ C)/β^N^	α^0^/α** + **β^0^/β^N^	4	0.003	0.658
-@-^SEA^/αα	Chinese ^G^γ + (^A^γδβ)^0^/β^N*^	α^0^/α** + **β^0^/β^N^	4	0.003	0.658
-@-^SEA^/αα	5'UTR + 43 to + 40 (− AAAC)/β^N*^	α^0^/α** + **β^+^/β^N^	8	0.006	1.316
α^CS^α/αα	IVS II-654 C > T/β^N^	α^+^/α** + **β^+^/β^N^	4	0.003	0.658
-α^4.2^/αα	CD 17 AAG > TAG [Lys > STOP]/β^N^	α^+^/α** + **β^0^/β^N^	3	0.002	0.493
-α^3.7^/αα	SEA-HPFH /β^N*^	α^+^/α** + **β^0^/β^N^	3	0.002	0.493
-α^3.7^/αα	CD 26 GAG > AAG [Glu > Lys]/β^N^	α^+^/α** + **β^+^/β^N^	3	0.002	0.493
-α^3.7^/αα	5'UTR + 43 to + 40 (− AAAC)/β^N*^	α^+^/α** + **β^+^/β^N^	6	0.004	0.987
-@-^THAI^/αα	CD 41/42 (− CTTT)/β^N^	α^0^/α** + **β^0^/β^N^	3	0.002	0.493
-@-^SEA^/αα	CD 26 GAG > AAG [Glu > Lys]/β^N^	α^0^/α** + **β^+^/β^N^	3	0.002	0.493
-@-^SEA^/αα	CD71-72 (+ A)/β^N^	α^0^/α** + **β^0^/β^N^	3	0.002	0.493
-@-^SEA^/αα	− 50 (G > A)/β^N*^	α^0^/α** + **β^+^/β^N^	3	0.002	0.493
α^WS^α/αα	CD 17 AAG > TAG [Lys > STOP]/β^N^	α^+^/α** + **β^0^/β^N^	3	0.002	0.493
α^WS^α/αα	− 28 (A > G)/β^N^	α^+^/α** + **β^+^/β^N^	3	0.002	0.493
-α^4.2^/αα	SEA-HPFH /β^N*^	α^+^/α** + **β^0^/β^N^	2	0.001	0.329
-α^4.2^/αα	− 50 (G > A)/β^N*^	α^+^/α** + **β^+^/β^N^	2	0.001	0.329
-α^3.7^/αα	CD27-28 (+ C)/β^N^	α^+^/α** + **β^0^/β^N^	2	0.001	0.329
-α^3.7^/αα	Chinese ^G^γ + (^A^γδβ)^0^/β^N*^	α^+^/α** + **β^0^/β^N^	2	0.001	0.329
-α^3.7^/αα	− 90 (C > T)/β^N*^	α^+^/α** + **β^+^/β^N^	2	0.001	0.329
-@-^SEA^/αα	CAP + 22 (G > A)/β^N*^	α^0^/α** + **β^+^/β^N^	2	0.001	0.329
-@-^SEA^/αα	− 29 (A > G)/β^N^	α^0^/α** + **β^+^/β^N^	2	0.001	0.329
-α^3.7^/-@-^SEA^	IVS II-654 C > T/β^N^	α^+^/α^0^** + **β^+^/β^N^	2	0.001	0.329
α^QS^α/αα	CD 41/42 (− CTTT)/β^N^	α^+^/α** + **β^0^/β^N^	2	0.001	0.329
α^CS^α/αα	CD 41/42 (− CTTT)/β^N^	α^+^/α** + **β^0^/β^N^	2	0.001	0.329
-α^4.2^/αα	CD71-72 (+ A)/β^N^	α^+^/α** + **β^0^/β^N^	1	0.001	0.164
-α^4.2^/αα	CD27-28 (+ C)/β^N^	α^+^/α** + **β^0^/β^N^	1	0.001	0.164
-α^4.2^/αα	Chinese ^G^γ + (^A^γδβ)^0^/β^N*^	α^+^/α** + **β^0^/β^N^	1	0.001	0.164
-α^4.2^/αα	5'UTR + 43 to + 40 (− AAAC)/β^N*^	α^+^/α** + **β^+^/β^N^	1	0.001	0.164
-α^3.7^/αα	CD 26 GAG > AAG [Glu > Lys]/β^N^	α^+^/α** + **β^+^/β^N^	1	0.001	0.164
-α^3.7^/αα	CD71-72 (+ A)/β^N^	α^+^/α** + **β^0^/β^N^	1	0.001	0.164
-α^3.7^/αα	CAP + 8 (C > T)/β^N*^	α^+^/α** + **β^+^/β^N^	1	0.001	0.164
-α^3.7^/αα	− 50 (G > A)/β^N*^	α^+^/α** + **β^+^/β^N^	1	0.001	0.164
-α^3.7^/αα	5'UTR + 43 to + 40 (− AAAC)/ − 28 (A > G)*	α^+^/α** + **β^+^/β^+^	1	0.001	0.164
α^QS^α/αα	CD 41/42 (− CTTT)/β^N^	α^+^/α** + **β^0^/β^N^	1	0.001	0.164
α^QS^α/αα	CD 43 (GAG > TAG)/β^N^	α^+^/α** + **β^0^/β^N^	1	0.001	0.164
-@-^SEA^/αα	Chinese ^G^γ + (^A^γδβ)^0^/β^N*^	α^0^/α** + **β^0^/β^N^	1	0.001	0.164
-@-^SEA^/αα	SEA-HPFH /β^N*^	α^0^/α** + **β^0^/β^N^	1	0.001	0.164
-@-^SEA^/αα	IVS-I-1 (G > T)/β^N^	α^0^/α** + **β^0^/β^N^	1	0.001	0.164
-@-^SEA^/αα	CD 126 GTG > GGG [Val > Gly]/β^N*^	α^0^/α** + **β^0^/β^N^	1	0.001	0.164
-@-^SEA^/αα	CD14-15 (+ G)/β^N^	α^0^/α** + **β^0^/β^N^	1	0.001	0.164
-@-^SEA^/αα	CD 43 (GAG > TAG)/β^N^	α^0^/α** + **β^0^/β^N^	1	0.001	0.164
-@-^SEA^/αα	Chinese ^G^γ + (^A^γδβ)^0^/ − 28 (A > G)*	α^0^/α** + **β^0^/β^+^	1	0.001	0.164
-@-^SEA^/αα	CAP + 8 (C > T)/β^N*^	α^0^/α** + **β^+^/β^N^	1	0.001	0.164
-@-^SEA^/αα	− 90 (C > T)/β^N*^	α^0^/α** + **β^0^/β^N^	1	0.001	0.164
-@-^SEA^/αα	− 72 (T > A)/β^N*^	α^0^/α** + **β^+^/β^N^	1	0.001	0.164
-α^4.2^/-α^4.2^	CD 41/42 (− CTTT)/β^N^	α^+^/α^+^** + **β^0^/β^N^	1	0.001	0.164
-α^4.2^/-α^4.2^	CD27-28 (+ C)/β^N^	α^+^/α^+^** + **β^0^/β^N^	1	0.001	0.164
-α^3.7^/-α^3.7^	CD 41/42 (− CTTT)/β^N^	α^+^/α^+^** + **β^0^/β^N^	1	0.001	0.164
-α^3.7^/-@-^SEA^	CD27-28 (+ C)/β^N^	α^+^/α^0^** + **β^0^/β^N^	1	0.001	0.164
-α^3.7^/-@-^SEA^	− 28 (A > G)/β^N^	α^+^/α^0^** + **β^+^/β^N^	1	0.001	0.164
Init CD ATG > A-G /αα	IVS II-654 C > T/β^N^	α^+^/α** + **β^+^/β^N^	1	0.001	0.164
α^WS^α/αα	CD 26 GAG > AAG [Glu > Lys]/β^N^	α^+^/α** + **β^+^/β^N^	1	0.001	0.164
α^QS^α/αα	CD 26 GAG > AAG [Glu > Lys]/β^N^	α^+^/α** + **β^+^/β^N^	1	0.001	0.164
α^QS^α/αα	CD 17 AAG > TAG [Lys > STOP]/β^N^	α^+^/α** + **β^0^/β^N^	1	0.001	0.164
α^CS^α/αα	CD 17 AAG > TAG [Lys > STOP]/β^N^	α^+^/α** + **β^0^/β^N^	1	0.001	0.164
α^CS^α/αα	− 50 (G > A)/β^N*^	α^+^/α** + **β^+^/β^N^	1	0.001	0.164
α^CS^α/αα	− 28 (A > G)/β^N^	α^+^/α** + **β^+^/β^N^	1	0.001	0.164
α^CS^α/αα	IVS II-654 C > T/β^N^	α^+^/α** + **β^+^/β^N^	1	0.001	0.164
α^CS^α/αα	Chinese ^G^γ + (^A^γδβ)^0^/β^N*^	α^+^/α** + **β^0^/β^N^	1	0.001	0.164
CD 30 -GAG [-Glu]/αα	Chinese ^G^γ + (^A^γδβ)^0^/β^N*^	α^+^/α** + **β^0^/β^N^	1	0.001	0.164
Total			608	0.446	100.000

*Indicates genotypes with rare mutations

### Allele frequency of α-thalassemia and β-thalassemia

**Fig. 2:**
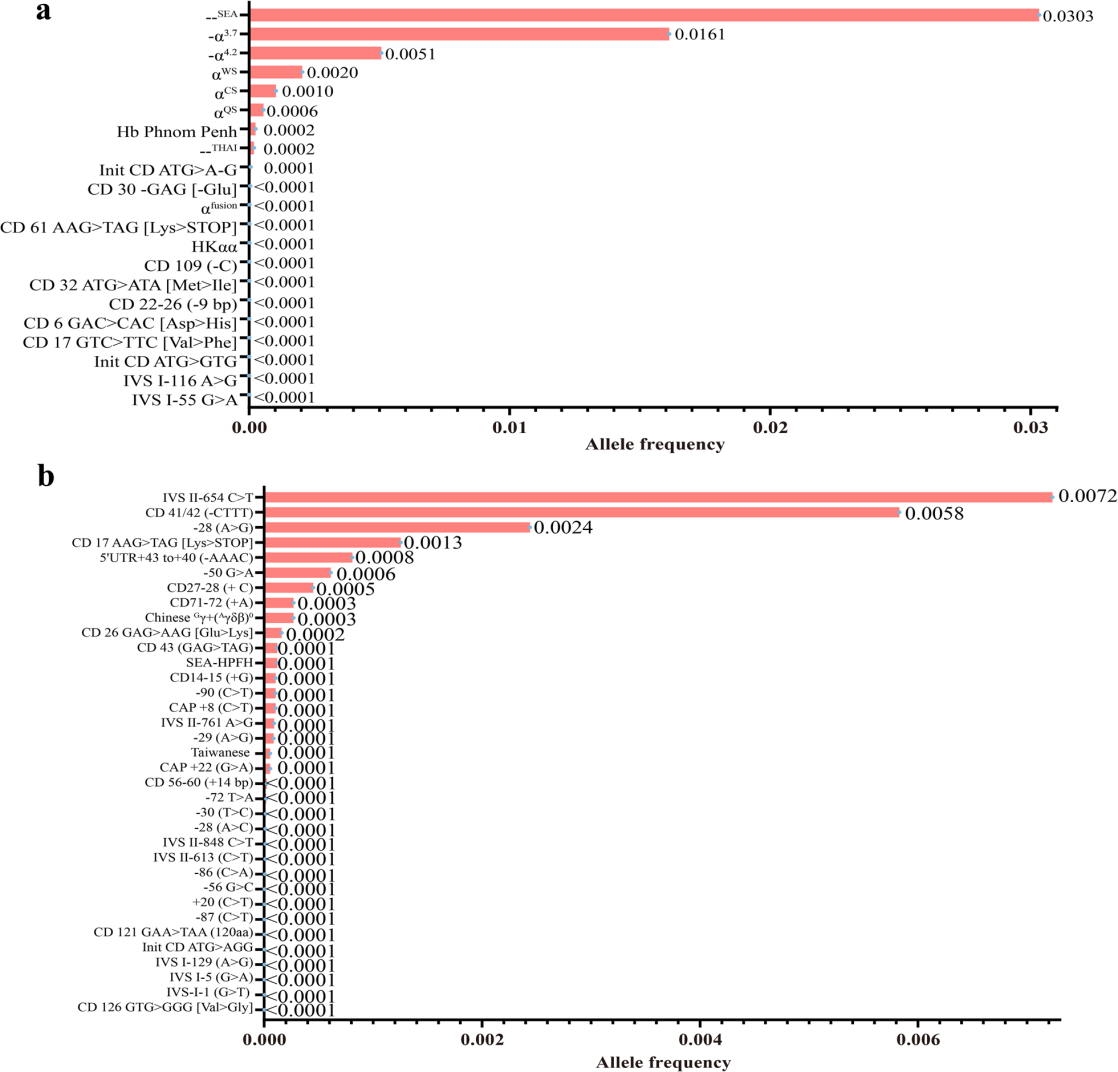
Thalassemia allele frequency distribution. α-thalassemia allele frequency distribution (**a**). β-thalassemia allele frequency distribution (**b**). Allele frequency = Number of alleles/total number of chromosomes investigated (136,312*2)

Figure 2 shows the allele frequency of different thalassemia mutations. Here, 21 α-gene mutations and 35 β-gene mutations were analyzed. The -@-^SEA^ allele was the allele most frequently mutated, with an allele frequency of 0.0303. Other mutations with high frequencies were -α^3.7^, -α^4.2^, α^WS^, α^CS^, and α^QS^, with allele frequencies of 0.0161, 0.0051, 0.0020, and 0.0010, respectively (Additional file 1: Table S3). Moreover, 15 rare α-mutations were identified. Of all the β-globin mutations, IVS II-654 C > T was the most frequent, with an allele frequency of 0.0072. Other high-frequency mutations included CD 41/42 (− CTTT), − 28 (A > G), CD 17 AAG > TAG [Lys > STOP], − 50 G > A, and 5'UTR + 43 to + 40 (− AAAC), with allele frequencies of 0.0058, 0.0024, 0.0013, and 0.0006, respectively (Additional file 1: Table S4).

### MCV and MCH values in positive samples

**Fig. 3:**
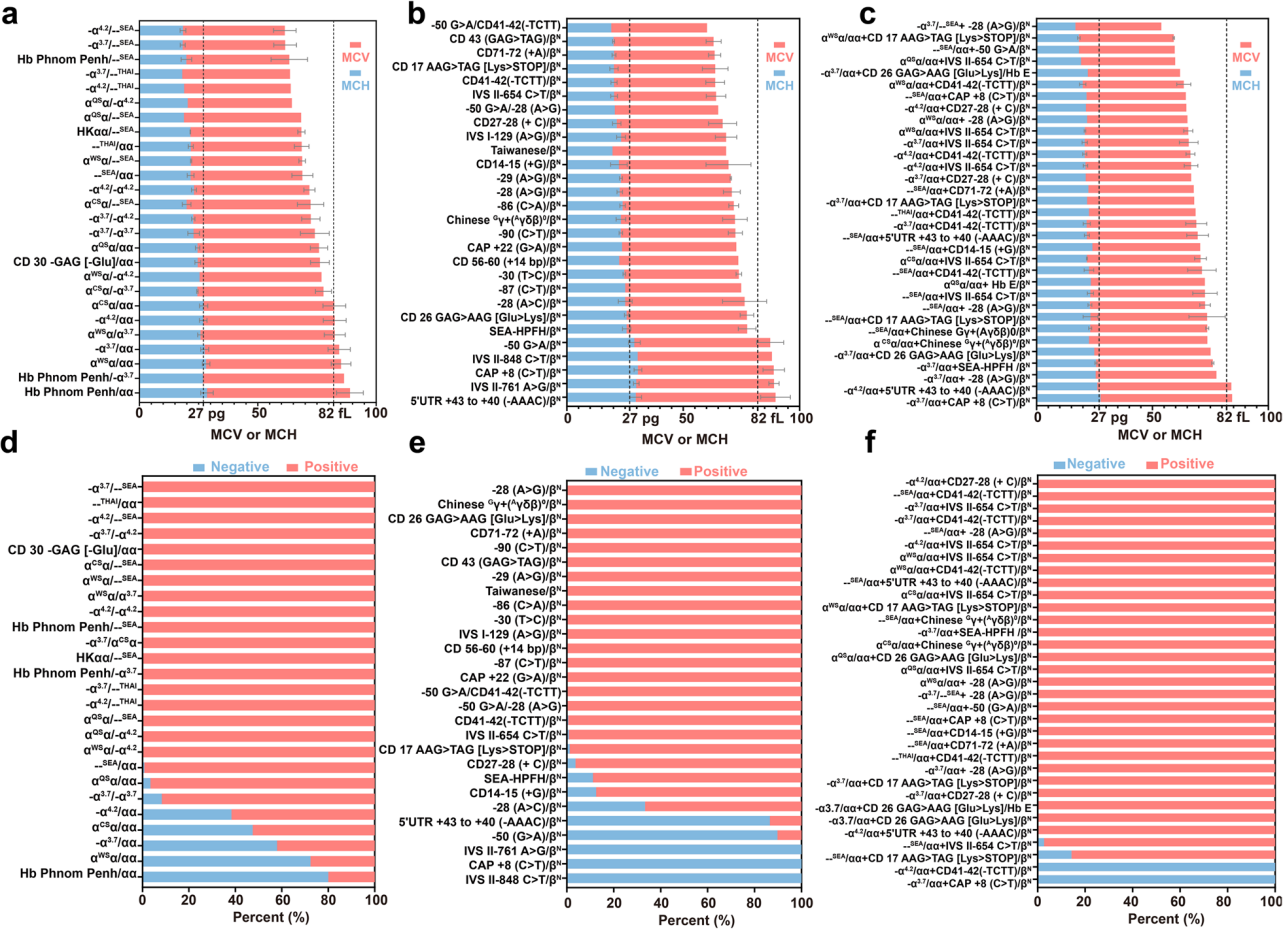
Hematological parameters of thalassemia populations and percentage of thalassemia subjects who passed blood cell screening. Hematological parameters of α-, β-, and α + β thalassemia population (**a**–**c**). Percentage of α-, β-, and α + β thalassemia subjects who passed blood cell screening (**d**–**f**). Negative: MCV ≥ 82 fL and MCH ≥ 27 pg. Positive: MCV < 82 fL and/or MCH < 27 pg

In this study, the relationship between the genotypes of α/β globin mutations and the characteristics of thalassemia MCV or MCH levels was also analyzed (Fig. 3). The level of MCV in most cases was lower than 82 fL, except for the genotypes Hb Phnom Penh/αα, α^WS^α/αα, -α^3.7^/αα, Hb Phnom Penh/-α^3.7^, 5'UTR + 43 to + 40 (− AAAC)/β^N^, IVS II-761 A > G/β^N^, CAP + 8 (C > T)/β^N^, IVS-II-848 C > T/β^N^, − 50 G > A/β^N^ and -α^3.7^/αα + CAP + 8 (C > T)/β^N^. However, the MCV values in individuals (1.5668%) with rare mutations were less than 82 fL, which emphasizes the importance of rare mutations in thalassemia carriers. It is worth mentioning that the results on MCH levels were highly consistent with those of MCV values. Moreover, most thalassemia carriers presented with abnormal MCV and MCH index values (MCV < 82 fL and/or MCH < 27 pg), while a few had normal values including subjects with the genotypes IVS-II-848 C > T/β^N^, IVS II-761 A > G/β^N^, and CAP + 8 (C > T)/β^N^. These results indicated that thalassemia carriers with these genotypes, especially those containing rare mutations, would be missed using routine hematological screening methods.

## Discussion

Thalassemia is a common genetic disease causing significant public health problems and social burdens in endemic areas [21, 22]. In recent years, the incidence of thalassemia has gradually decreased with the improvement and widespread popularization of genetic counseling and prenatal diagnosis (PND) technologies [23, 24]. However, a high prevalence of thalassemia has still been reported in southern China due to the lack of PND and genetic counseling [9, 18]. The overall prevalence of α-thalassemia, β-thalassemia, and α + β-thalassemia in this study was 7.880%, 2.210%, and 0.480%, respectively [25]. Ganzhou is the southernmost city of Jiangxi Province, central China, and it is adjacent to Guangdong Province, which had one of the highest incidence rates of thalassemia in China [26]. Therefore, investigating the genotype and distribution of thalassemia in Ganzhou city is of great significance for providing a theoretical basis for PND and genetic counseling.

In this study, NGS was applied for large-scale population screening to assess the frequency of thalassemia carriers among people in the Gannan region. The results demonstrated the great heterogeneity and widespread spectrum of thalassemia in the Gannan population. The overall frequency of thalassemia was 14.545%, which was significantly higher than that (10.570%) nationwide [25]. Furthermore, the incidence of α-thalassemia (10.489%) was significantly higher than that of β-thalassemia (3.610%) in the Gannan region (*p* < 0.05), which was in accordance with previous studies [14]. These results indicate that thalassemia is a serious public health problem in the Gannan region. It is interesting to note that the prevalence rate of thalassemia decreased from the south to the north in this province. The region with the highest prevalence was Dingnan (18.317%), followed by Xunwu (17.723%). One reason for this trend may have been that Dingnan and Xunwu are situated in southeastern Jiangxi Province at the junction of Fujian, Guangdong, and Jiangxi Provinces. The vast majority of the residents in Dingnan are Hakka people, who have been previously reported to have a high prevalence of thalassemia [27, 28]. More importantly, with the application of NGS to a large population, our data more accurately reflect the prevalence of thalassemia and the distribution of rare thalassemia genotypes in the Gannan region.

The detection rate (10.489%) for α-thalassemia in this study was significantly higher than that previously reported (7.190%) in the Gannan region or (2.600%) in Jiangxi [13, 14]. We attribute the differences to different genetic screening methods, and sample sizes between these two studies. In addition, we identified 40 distinct α-thalassemia genotypes with 21 different variations, in which, αα/-@-^SEA^ was the most common subtype, with a remarkable proportion of 54.105%, followed by -^3.7^/αα (28.011%) and -α^4.2^/αα (8.687%), which was consistent with previous reports [14, 29]. Apart from these common variation types, other variations with rare or novel mutations were also identified. Hb Phnom Penh, a rare variant caused by the insertion of an ATC (for isoleucine) between codons 117 and 118, was identified as a hotspot for nucleotide insertions within exon 3 of the α1-globin gene. It was first reported in the Cambodian population [30] but has been rarely reported in the mainland of China or Taiwan province [31, 32]. -@-^THAI^ (NC_000016.9: g.199800_233300del), which has been reported in southern China except for Jiangxi Province was also detected in this study [33–35]. Furthermore, we also detected other rare genotypes that have not been reported in Jiangxi Province, including CD 30 -GAG [-Glu], Init CD ATG > A-G, and α^fusion^. These novel findings greatly enrich the database of known thalassemia alleles in the Gannan region.

A total of 35 β-thalassemia variations with 42 genotypes that have not been reported in our previous study using RDB gene chips were identified in this cohort, our results suggested that NGS was preferable to RDB gene chip for the screening of rare variants [14]. The prevalence of β-thalassemia (3.36%) in this study was much higher than the reported average of 2.21% in China [25]. In addition to conventional β-thalassemia mutants, rare deletion variants, including Chinese ^G^γ + (^A^γδβ)^0^, SEA-HPFH, and Taiwanese deletion, were also detected. Regarding β-thalassemia genotypes, IVS II-654 C > T/β^N^ and CD 41/42 (− CTTT)/β^N^ were the two most frequently detected β-thalassemia subtypes, accounting for 35.257% and 28.368%, respectively. The ranking order of the two major mutations also was IVS II-654 C > T and CD 41/42 (− CTTT), which agreed with our previous observations [14]. It was interesting to note that these results were identical to those of the Hakka population in Meizhou, Guangdong Province [27, 29], and these results implied that the prevalence of β-thalassemia and its genotype distribution were geographically associated. In addition to the higher detection rate, our study also detected some rare β-thalassemia mutations that had not been reported previously, such as − 50 G > A and 5'UTR + 43 to + 40 (− AAAC), which accounted for 11.339% of all β-thalassemia genotypes.

Unexpectedly, two mutant homozygotes (− 28 (A > G)/ − 28 (A > G)) and nine compound heterozygotes were identified in this study, and the hematological parameters of the affected individuals were typical of thalassemia (microcytic hypochromic anemia). Moreover, compound heterozygotes included common mutations (− 28 (A > G), CD 41/42 (− CTTT), IVS II-654 C > T, CD 17 AAG > TAG [Lys > STOP], and compound rare mutations (Chinese ^G^γ + (^A^γδβ)^0^, − 50 G > A, 5'UTR + 43 to + 40 (− AAAC), CAP + 8 (C > T)). Undoubtedly, the application of conventional thalassemia genetic testing methods will not be able to accurately determine the genotypes of these populations.

With the development of NGS techniques in recent years, NGS has emerged as a powerful and cheaper tool for prenatal screening [18, 36]. To date, several studies have applied NGS for the study of thalassemia and have made great progress [19, 37]. In our study, high throughput thalassemia screening was conducted at $10 per sample. A total of 56 thalassemia mutations were identified, including 48 rare mutations. Traditional detection methods, such as RDB and Gap-PCR, can only detect 23 mutations [20], and therefore miss the remaining 33 mutations. In other words, 4.010% (795/19,827) of the population will be missed or misdiagnosed using traditional screening methods. Traditionally, RBC analysis combined with hemoglobin electrophoresis and clinical manifestation description is commonly used for preliminary screening of thalassemia. Then PCR or genome sequencing is used to confirm positive cases before diagnosis [1]. Limited by the low sensitivity of hematological analysis and the disadvantages of PCR, a large number of novel or rare thalassemia variations would be missed or misdiagnosed using traditional screening methods. To fill this gap, our findings suggest that NGS can effectively identify new mutations and reduce the rate of misdiagnosis.

Recently, third-generation sequencing (TGS) has been emerging as a fancy method to identify thalassemia mutations in GC-rich and high homology sequences, as well as complex structural mutations. However, it is expensive and time-consuming, which limits its availability in all diagnostic laboratories. In this regard, NGS-based thalassemia screening can benefit a large population with acceptably high accuracy and relatively affordable cost.

In summary, our study was the first to apply NGS to comprehensively analyze thalassemia in a large population of the Gannan region, Jiangxi Province. We demonstrated a high genetic diversity and a high prevalence of thalassemia in this region, which will be of great significance for the prevention and control of thalassemia in Gannan and other high-prevalence areas. More importantly, the identification of rare and novel variations highlighted the necessity and significance of choosing NGS for thalassemia screening in large populations.

### Supplementary Information

**Additional file 1.** Supplementary tables.

## Data Availability

The datasets used and analyzed during the current study are available from the corresponding author upon reasonable request.

## Abbreviations

DNA: Deoxyribonucleic acid
EDTA-K_2_: Ethylene diamine tetraacetic acid K_2_
MCH: Mean corpuscular hemoglobin
MCV: Mean corpuscular volume
NGS: Next-generation sequencing
PCR: Polymerase chain reaction
PND: Prenatal diagnosis
RBC: Red blood cell
RDB: Reverse dot blot
