# Supplementary material for: Next-generation sequencing analysis of the molecular spectrum of thalassemia in Southern Jiangxi, China
Source: Hum Genomics. 2023 Aug 17;17:77. doi: 10.1186/s40246-023-00520-5 (PMC10436446; doi:10.1186/s40246-023-00520-5)
Supplement: Supplementary file 1 — Additional file 1. Supplementary tables. [file 40246_2023_520_MOESM1_ESM.docx]

*Title Page for Human Genomics*

**Next-generation sequencing analysis of the molecular spectrum of thalassemia in Southern Jiangxi, China**

*Tong Yang*^a,b^^*, Xuemei Luo^c^^*, *Yanqiu Liu^d^^, Min Lin^e^, Qinfei Zhao^b^*, *Wenqian Zhang ^f,g^, Zhigang Chen ^f,g^, Minghua Dong^h^, Junli Wang^i^, Qi Wang ^a^, Xiaokang Zhang^h*^, Tianyu Zhong^a,b*^*

^a^The First School of Clinical Medicine, Gannan Medical University, Ganzhou, China

^b^Laboratory Medicine, First Affiliated Hospital of Gannan Medical University, Ganzhou, China

^c^Ganzhou Municipal Health Commission, Ganzhou, China

^d^Department of Medical Genetics, Jiangxi Maternal and Child Health Hospital, Nanchang, China

^e^School of Food Engineering and Biotechnology, Hanshan Normal University, Chaozhou, China

^f^ BGI Genomics, BGI-Shenzhen, Shenzhen, China

^g^BGI-Wuhan Clinical Laboratories, BGI-Shenzhen, Wuhan, China

^h^School of public health and health management, Gannan Medical University, Ganzhou, China

*^i^*Affiliated hospital of Youjiang Medical University for Nationalities, Baise, China.

^^^These authors contributed equally to this work.

^*^Address correspondence to the authors at:

Xiaokang Zhang- Department of Preventive Medicine, Gannan Medical University, Ganzhou, Jiangxi 341000, China; Email: zhangxiaokaju@163.com

Tianyu Zhong- Laboratory Medicine, First Affiliated Hospital of Gannan Medical University, Ganzhou, Jiangxi 341000, China, Tel: 86-0759-8689240, Email: zhongtianyu@gmail.com

**Supplemental Table S1.** Thalassemia genotype definition.

HGVS, human genome variation society. N/A, Not Applicable

| **Common Nane** | **HGVS Name** | **Gene** | **Common or Rare** |
| --- | --- | --- | --- |
| -α^3.7^ | NG_000006.1:g.34247_38050del | α1,α2 | Common |
| -α^4.2^ | NC_000016.10:g.169818_174075del | α2 | Common |
| -^SEA^ | NC_000016.10:g.165401_184701del | α1,α2 | Common |
| CD 142 (TAA>CAA) >172aa (α^CS^α) | HBA2: c.427 T>C | α2 | Common |
| CD 125 CTG>CCG [Leu>Pro](α^QS^α) | HBA2: c.377 T>C | α2 | Common |
| CD 122 CAC>CAG [His>Gln] (α^WS^α) | HBA2: c.369 C> G | α2 | Common |
| --^THAI^ | NC_000016.9:g.199800_233300del | α1,α2 | Rare |
| CD 117/118 +ATC [+Ile] (Hb Phnom Penh) | HBA1: HBA1:c.354_355insATC | α1 | Rare |
| Init CD ATG>A-G | HBA1: c. 2 delT | α1 | Rare |
| CD 30 -GAG [-Glu] | HBA2: c.91_93delGAG | α2 | Rare |
| CD 61 AAG>TAG [Lys>STOP] | HBA2: c.184A>T | α2 | Rare |
| CD 109 (-C) | HBA1: c.328delC | α1 | Rare |
| HKαα | N/A | α2, α1 | Rare |
| CD 22-26 (-9 bp) | HBA2: c.69_77delCGAGTATGG | α2 | Rare |
| CD 32 ATG>ATA [Met>Ile] | HBA1: c.99G>A | α1 | Rare |
| CD 6 GAC>CAC [Asp>His] | HBA2: c.19G>C | α2 | Rare |
| CD 17 GTC>TTC [Val>Phe] | HBA2: c.52G>T | α2 | Rare |
| Init CD ATG>GTG | HBA1: c.1A>G | α1 | Rare |
| IVS I-116 A>G | HBA1: c.96-2A>G | α1 | Rare |
| IVS I-55 G>A | HBA2: c.95+55G>A | α2 | Rare |
| CD41-42(-TCTT) | HBB:c.126_129delCTTT | β^0^ | Common |
| IVS-II-654 (C>T) | HBB: c.316-197C>T | β^+^ | Common |
| CD 17 AAG>TAG [Lys>STOP] | HBB: c.52A>T | β^0^ | Common |
| -28 (A>G) | HBB:c.-78A>G | β^+^ | Common |
| -28 (A>C) | HBB:c.-78A>C | β^+^ | Common |
| CD 26 GAG>AAG [Glu>Lys] | HBB: c.79G>A | β^+^ | Common |
| CD71-72 (+A) | HBB: c.216_217insA | β^0^ | Common |
| CD 43 (GAG>TAG) | HBB:c.130G>T | β^0^ | Common |
| -29 (A>G) | HBB:c.-79A>G | β^+^ | Common |
| CD14-15 (+G) | HBB:c.45_46insG | β^0^ | Common |
| CD27-28 (+ C) | HBB:c.84_85insC | β^0^ | Common |
| -30 (T>C) | HBB:c.-80T>C | β^0^ | Common |
| IVS I-1 (G>T) | HBB:c.92+1G>T | β^0^ | Common |
| IVS I-5 (G>A) | HBB:c.92+5G>A | β^+^ | Common |
| Init CD ATG>AGG | HBB: c.2T>G | β^0^ | Common |
| -50 G>A | HBB: c.-100G>A | β^+^ | Rare |
| 5'UTR+43 to+40 (-AAAC) | HBB: c.-11_-8delAAAC | β^+^ | Rare |
| Chinese ^G^γ+(^A^γδβ)^0^ | NC_000011.9: g.5191148_ 5270051del | β^0^ | Rare |
| SEA-HPFH | NC_000011.10:g.5201647_5229059del | β^0^ | Rare |
| -90 (C>T) | HBB: c.-140C>T | β^+^ | Rare |
| IVS II-761 A>G | HBB: c.316-90A>G | β^0^ | Rare |
| CAP +8 (C>T) | HBB: c.-43C>T | β^+^ | Rare |
| Taiwanese | NC_000011.9: g.5247493_5248849del | β^0^ | Rare |
| CAP +22 (G>A) | HBB: c.-29G>A | β^+^ | Rare |
| -72 T>A | HBB:c.-122T>A | β^+^ | Rare |
| CD 56-60 (+14 bp) | HBB:c.170_183dup | β^0^ | Rare |
| IVS II-848 C>T | HBB: c.316-3C>T | β^+^ | Rare |
| -86 (C>A) | HBB: c.-136C>A | β^+^ | Rare |
| IVS II-613 (C>T) | HBB.c.316-238C>T | β^+^ | Rare |
| -56 G>C | HBB:c.-106G>C | β^+^ | Rare |
| +20 (C>T) | HBB:c.-31C>T | β^+^ | Rare |
| -87 (C>T) | HBB:c.-137C>T | β^+^ | Rare |
| CD 121 GAA>TAA (120aa) | HBB:c.364G>T | β^0^ | Rare |
| IVS I-129 (A>G) | HBB: c.93-2A>G | β^0^ | Rare |
| CD 126 GTG>GGG [Val>Gly] | HBB: c.380T>G | β^0^ | Rare |

**Supplemental Table S2**. Prevalence rate of α-thalassemia, β-thalassemia, and combined α-/β-thalassemia in Southern Jiangxi, China.

|  | **Non-thalassemia**  **（%）** | **α-thalassemia (%)** | **β-thalassemia (%)** | **Combined α-/β- thalassemia (%)** | **Total** | **Frequency**  **(%)** |
| --- | --- | --- | --- | --- | --- | --- |
| **Total N (%)** | 116,485(85.455) | 14298(10.489) | 4921(3.610) | 608(0.446) | 136,312 | 14.545 |
| **Region** |  |  |  |  |  |  |
| Ningdu | 9,716(89.515) | 821(7.564) | 296(2.727) | 21(0.193) | 10,854 | 10.485 |
| Shicheng | 4,566(88.471) | 461(8.932) | 130(2.519) | 4(0.078) | 5,161 | 11.529 |
| Zhanggong | 7,919(87.089) | 836(9.194) | 312(3.431) | 26(0.286) | 9,093 | 12.911 |
| Ganxian | 7,863(86.683) | 888(9.789) | 286(3.153) | 34(0.375) | 9,071 | 13.317 |
| Xingguo | 9,045(86.431) | 1010(9.651) | 363(3.469) | 47(0.449) | 10,465 | 13.569 |
| Yudu | 14,438(86.352) | 1608(9.617) | 605(3.618) | 69(0.413) | 16,720 | 13.648 |
| Ruijin | 9,498(85.667) | 1156(10.427) | 388(3.500) | 45(0.406) | 11,087 | 14.333 |
| Shangyou | 3,204(84.942) | 419(11.108) | 128(3.393) | 21(0.557) | 3,772 | 15.058 |
| Dayu | 2,256(84.589) | 304(11.399) | 90(3.375) | 17(0.637) | 2,667 | 15.411 |
| Nankang | 9,125(84.429) | 1269(11.741) | 361(3.340) | 53(0.490) | 10,808 | 15.571 |
| Huichang | 7,827(84.252) | 1058(11.389) | 359(3.864) | 46(0.495) | 9,290 | 15.748 |
| Anyuan | 5,316(83.703) | 708(11.148) | 290(4.566) | 37(0.583) | 6,351 | 16.297 |
| Xinfeng | 10,023(83.491) | 1426(11.878) | 486(4.048) | 70(0.583) | 12,005 | 16.509 |
| Quannan | 1,852(83.424) | 252(11.351) | 99(4.459) | 17(0.766) | 2,220 | 16.576 |
| Longnan | 4,313(83.311) | 621(11.995) | 208(4.018) | 35(0.676) | 5,177 | 16.689 |
| Chongyi | 2,423(82.894) | 355(12.145) | 126(4.311) | 19(0.650) | 2,923 | 17.106 |
| Xunwu | 5,130(82.278) | 809(12.975) | 260(4.170) | 36(0.577) | 6,235 | 17.722 |
| Dingnan | 1,971(81.683) | 297(12.308) | 134(5.553) | 11(0.456) | 2,413 | 18.317 |
| **Sex** |  |  |  |  |  |  |
| Male | 60,676(85.347) | 7,537(10.602) | 2,561(3.602) | 319(0.449) | 71,093 | 14.653 |
| Female | 55,809(85.571) | 6,761(10.367) | 2,360(3.619) | 289(0.443) | 65,219 | 14.429 |

**Supplemental Table S3**. Allele frequency of α-thalassemia among the 136,312 subjects in Southern Jiangxi, China. Allele frequency = Number of alleles / total number of chromosomes investigated (136,312*2).

| **Mutation** | **Allele (n)** | **Frequency** | **Constituent ratio (%)** |
| --- | --- | --- | --- |
| --^SEA^ | 8,267 | 0.0303 | 54.3631 |
| -α^3.7^ | 4,396 | 0.0161 | 28.9077 |
| -α^4.2^ | 1,385 | 0.0051 | 9.1076 |
| α^WS^ | 557 | 0.0020 | 3.6628 |
| α^CS^ | 280 | 0.0010 | 1.8413 |
| α^QS^ | 150 | 0.0006 | 0.9864 |
| Hb Phnom Penh | 67 | 0.0002 | 0.4406 |
| --^THAI^ | 51 | 0.0002 | 0.3354 |
| Init CD ATG>A-G | 17 | 0.0001 | 0.1118 |
| CD 30 -GAG [-Glu] | 11 | <0.0001 | 0.0723 |
| CD 61 AAG>TAG [Lys>STOP] | 4 | <0.0001 | 0.0263 |
| CD 109 (-C) | 3 | <0.0001 | 0.0197 |
| HKαα | 4 | <0.0001 | 0.0263 |
| CD 22-26 (-9 bp) | 2 | <0.0001 | 0.0132 |
| α^fusion^ | 5 | <0.0001 | 0.0329 |
| CD 32 ATG>ATA [Met>Ile] | 3 | <0.0001 | 0.0197 |
| CD 6 GAC>CAC [Asp>His] | 1 | <0.0001 | 0.0066 |
| CD 17 GTC>TTC [Val>Phe] | 1 | <0.0001 | 0.0066 |
| Init CD ATG>GTG | 1 | <0.0001 | 0.0066 |
| IVS I-116 A>G | 1 | <0.0001 | 0.0066 |
| IVS I-55 G>A | 1 | <0.0001 | 0.0066 |
| **Total** | **15,207** | **0.0558** | **100.0000** |

| **Mutation** | **Allele (n)** | **Frequency** | **Constituent ratio (%)** |
| --- | --- | --- | --- |
| IVS II-654 C>T | 1,972 | 0.0072 | 35.6021 |
| CD 41/42 (-CTTT) | 1,589 | 0.0058 | 28.6875 |
| -28 (A>G) | 665 | 0.0024 | 12.0058 |
| CD 17 AAG>TAG [Lys>STOP] | 342 | 0.0013 | 6.1744 |
| -50 (G>A) | 167 | 0.0006 | 3.0150 |
| 5'UTR+43 to+40 (-AAAC) | 221 | 0.0008 | 3.9899 |
| CD27-28 (+ C) | 123 | 0.0005 | 2.2206 |
| CD71-72 (+A) | 74 | 0.0003 | 1.3360 |
| Chinese ^G^γ+(^A^γδβ)^0^ | 74 | 0.0003 | 1.3360 |
| CD 43 (GAG>TAG) | 33 | 0.0001 | 0.5958 |
| CD14-15 (+G) | 29 | 0.0001 | 0.5236 |
| SEA-HPFH | 33 | 0.0001 | 0.5958 |
| -90 (C>T) | 29 | 0.0001 | 0.5236 |
| IVS II-761 A>G | 25 | 0.0001 | 0.4513 |
| -29 (A>G) | 24 | 0.0001 | 0.4333 |
| CAP +8 (C>T) | 29 | 0.0001 | 0.5236 |
| Taiwanese | 15 | 0.0001 | 0.2708 |
| CAP +22 (G>A) | 15 | 0.0001 | 0.2708 |
| CD 26 GAG>AAG [Glu>Lys] | 44 | 0.0002 | 0.7944 |
| -72 T>A | 5 | <0.0001 | 0.0903 |
| CD 56-60 (+14 bp) | 7 | <0.0001 | 0.1264 |
| -28 (A>C) | 3 | <0.0001 | 0.0542 |
| -30 (T>C) | 4 | <0.0001 | 0.0722 |
| IVS II-848 C>T | 3 | <0.0001 | 0.0542 |
| -86 (C>A) | 2 | <0.0001 | 0.0361 |
| IVS II-613 (C>T) | 3 | <0.0001 | 0.0542 |
| -56 (G>C) | 1 | <0.0001 | 0.0181 |
| +20 (C>T) | 1 | <0.0001 | 0.0181 |
| -87 (C>T) | 1 | <0.0001 | 0.0181 |
| CD 121 GAA>TAA (120aa) | 1 | <0.0001 | 0.0181 |
| Init CD ATG>AGG | 1 | <0.0001 | 0.0181 |
| IVS I-129 (A>G) | 1 | <0.0001 | 0.0181 |
| IVS I-5 (G>A) | 1 | <0.0001 | 0.0181 |
| IVS I-1 (G>T) | 1 | <0.0001 | 0.0181 |
| CD 126 GTG>GGG [Val>Gly] | 1 | <0.0001 | 0.0181 |
| **Total** | **5,539** | **0.0203** | **100.0000** |

**Supplemental Table S4**. Allele frequency of β-thalassemia among the 136,312 subjects in Southern Jiangxi, China. Allele frequency = Number of alleles / total number of chromosomes investigated (136,312*2).
